# A Recombinant Fusion Toxin Based on Enzymatic Inactive C3bot1 Selectively Targets Macrophages

**DOI:** 10.1371/journal.pone.0054517

**Published:** 2013-01-21

**Authors:** Lydia Dmochewitz, Christina Förtsch, Christian Zwerger, Martin Vaeth, Edward Felder, Markus Huber-Lang, Holger Barth

**Affiliations:** 1 Institute of Pharmacology and Toxicology, University of Ulm Medical Center, Ulm, Germany; 2 Institute of Pathology, Julius-Maximilians University, Würzburg, Germany; 3 Institute of General Physiology, University of Ulm, Ulm, Germany; 4 Institute of Traumatology, Hand- and Reconstructive Surgery, University of Ulm Medical Center, Ulm, Germany; Institute Pasteur, France

## Abstract

**Background:**

The C3bot1 protein (∼23 kDa) from *Clostridium botulinum* ADP-ribosylates and thereby inactivates Rho. C3bot1 is selectively taken up into the cytosol of monocytes/macrophages but not of other cell types such as epithelial cells or fibroblasts. Most likely, the internalization occurs by a specific endocytotic pathway via acidified endosomes.

**Methodology/Principal Findings:**

Here, we tested whether enzymatic inactive C3bot1E174Q serves as a macrophage-selective transport system for delivery of enzymatic active proteins into the cytosol of such cells. Having confirmed that C3bot1E174Q does not induce macrophage activation, we used the actin ADP-ribosylating C2I (∼50 kDa) from *Clostridium botulinum* as a reporter enzyme for C3bot1E174Q-mediated delivery into macrophages. The recombinant C3bot1E174Q-C2I fusion toxin was cloned and expressed as GST-protein in *Escherichia coli*. Purified C3bot1E174Q-C2I was recognized by antibodies against C2I and C3bot and showed C2I-specific enzyme activity *in vitro*. When applied to cultured cells C3bot1E174Q-C2I ADP-ribosylated actin in the cytosol of macrophages including J774A.1 and RAW264.7 cell lines as well as primary cultured human macrophages but not of epithelial cells. Together with confocal fluorescence microscopy experiments, the biochemical data indicate the selective uptake of a recombinant C3-fusion toxin into the cytosol of macrophages.

**Conclusions/Significance:**

In summary, we demonstrated that C3bot1E174Q can be used as a delivery system for fast, selective and specific transport of enzymes into the cytosol of living macrophages. Therefore, C3-based fusion toxins can represent valuable molecular tools in experimental macrophage pharmacology and cell biology as well as attractive candidates to develop new therapeutic approaches against macrophage-associated diseases.

## Introduction

Bacterial exotoxins, which act as enzymes in the cytosol of mammalian cells, are internalized by receptor-mediated endocytosis and exploit vesicular trafficking pathways of their target cells to deliver their enzyme moiety into the host cell cytosol (for review see [1], [2], [3], [4], [5]). Because the toxin uptake has no adverse effects on cells until the enzyme domain is released into the cytosol, enzyme deficient toxins or toxin fragments were exploited as molecular Trojan horses in experimental pharmacology and cell biology to deliver foreign proteins into the cytosol of mammalian cells (for review see [6], [7], [8]).

We have reported earlier that the Rho-ADP-ribosylating C3 ADP-ribosyltransferases (∼23 kDa) from *C. botulinum* (C3bot1) [9], [10], [11] and *C. limosum* [12] are efficiently taken up into the cytosol of monocytes/macrophages within 3 h resulting in mono-ADP-ribosylation and inactivation of Rho and thereby in a characteristic change of cell morphology [13]. Importantly, the C3 proteins are not taken up into the cytosol of other cell types such as epithelial cells and fibroblasts under comparable experimental conditions [13]. Most likely, both C3 proteins, as well as the enzymatic inactive C3bot1E174Q protein [14] are taken up into the cytosol of monocytes/macrophages by a specific endocytotic mechanism via acidified endosomal vesicles [13]. However, in contrast to the wild-type C3 proteins, internalized C3bot1E174Q has no effects on the morphology and viability of monocytes/macrophages [13]. Therefore C3bot1E174Q should represent an ideal carrier for specific and targeted delivery of enzymatic active fusion toxins into the cytosol of monocytes/macrophages.

In the present proof of principle study, the actin ADP-ribosylating C2I [15], [16] was used as a reporter enzyme. C2I, the enzyme component of the binary C2 toxin from *C. botulinum* [17], [18], [19], is not internalized into cells in the absence of the separate transport component C2IIa [5], [17], . During the normal uptake of C2 toxin, C2IIa forms a complex with C2I [21] and mediates the binding of the C2IIa/C2I complex to a carbohydrate receptor present on all mammalian cell types tested so far [22], [23], [24]. After receptor-mediated endocytosis of the toxin complex [25], [26], C2IIa forms pores in the membranes of acidified early endosomes and C2I translocates through these pores from the lumen of acidified endosomal vesicles into the cytosol [27], [28], [29], [30], [31]. Translocation of C2I is facilitated by the host cell chaperone Hsp90 [32] and the protein folding helper enzymes cyclophilin A [33] and FK506 binding protein 51 [34]. In the cytosol C2I mono-ADP-ribosylates actin what results in depolymerization of actin filaments [35], [36], [37].

Here, the cloning, purification and biochemical characterization of the recombinant fusion toxin C3bot1E174Q-C2I is reported. Biochemical and microscopic approaches demonstrate that the enzymatic active fusion toxin is efficiently taken up into the cytosol of macrophages but not epithelial cells.

## Results

### Cloning, Purification and Characterization of the C3bot1E174Q-C2I Fusion Toxin

**Figure 1:**
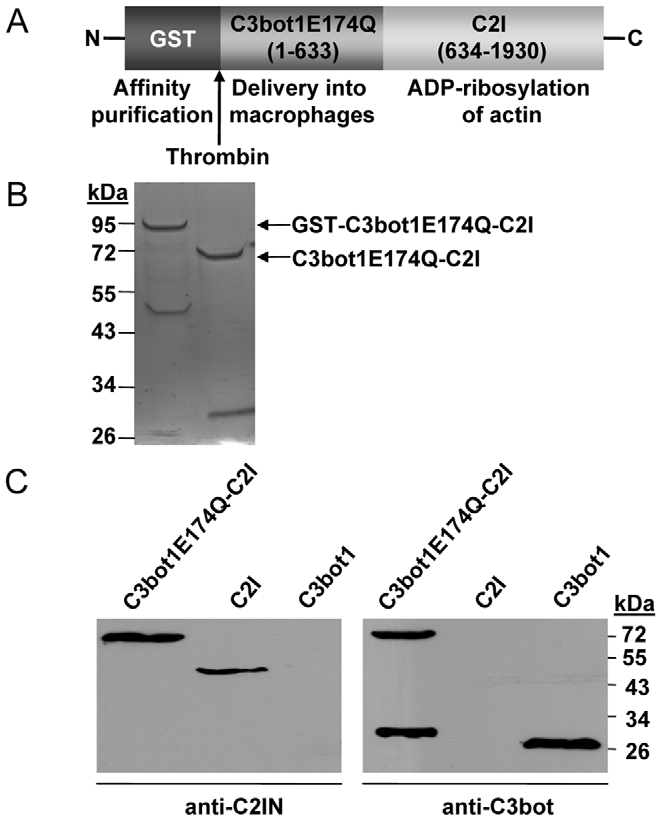
Characterization of C3bot1E174Q-C2I. *A.* The recombinant fusion toxin C3bot1E174Q-C2I is depicted. *B.* Coomassie blue staining of GST-C3bot1E174Q-C2I (left lane) and C3bot1E174Q-C2I (right lane) after SDS-PAGE. GST-C3bot1E174Q-C2I was expressed in *E. coli* and GST was cleaved off with thrombin. *C.* Western blot analysis of C3bot1E174Q-C2I. C3bot1E174Q-C2I (100 ng) as well as C2I (100 ng) and C3 (100 ng) for control were subjected to SDS-PAGE, blotted and proteins were detected with specific antibodies against C2I and C3bot.

**Figure 2:**
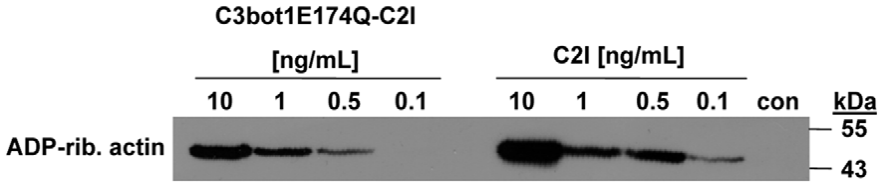
C3bot1E174Q-C2I ADP-ribosylates actin *in vitro*. Lysate from J774A.1 cells (20 µg) was either left untreated for control (con) or incubated for 10 min at 37°C with either C3bot1E174Q-C2I (0.1 ng/mL, 0.5 ng/mL, 1 ng/mL, 10 ng/mL) or C2I (0.1 ng/mL, 0.5 ng/mL, 1 ng/mL, 10 ng/mL) in the presence of biotin-NAD^+^ (10 µM). Samples were subjected to SDS-PAGE, blotted and biotinylated (i.e. ADP-ribosylated) actin was detected with streptavidin-peroxidase.

C3bot1E174Q-C2I was expressed as a GST-fusion protein in *E. coli* (Fig. 1A). C3bot1E174Q-C2I was purified by affinity chromatography with glutathion-sepharose as described in Materials and Methods. The identity of this fusion toxin was confirmed by SDS-PAGE (Fig. 1B) and Western blotting with specific antibodies against C3bot and C2I (Fig. 1C). *In vitro* ADP-ribosylation of actin was performed to test whether C3bot1E174Q-C2I shows the expected C2I-specific enzymatic activity. To this end, whole J74A.1 cell lysate was incubated with biotin-NAD^+^ as co-substrate in the presence of C3bot1E174Q-C2I (Fig. 2). As a positive control the actin ADP-ribosylating C2I was used as an enzyme. C2I covalently transfers biotin-ADP-ribose onto actin which can be detected with streptavidin-peroxidase by Western blotting. As shown in Fig. 2, actin was ADP-ribosylated by C2I as well as C3bot1E174Q-C2I, clearly indicating that the C3bot1E174Q-C2I fusion toxin was active *in vitro* and showed the C2I-specific ADP-ribosyltransferase activity.

### C3bot1E174Q-C2I ADP-ribosylates Actin in the Cytosol of Macrophages but not Epithelial Cells

**Figure 3:**
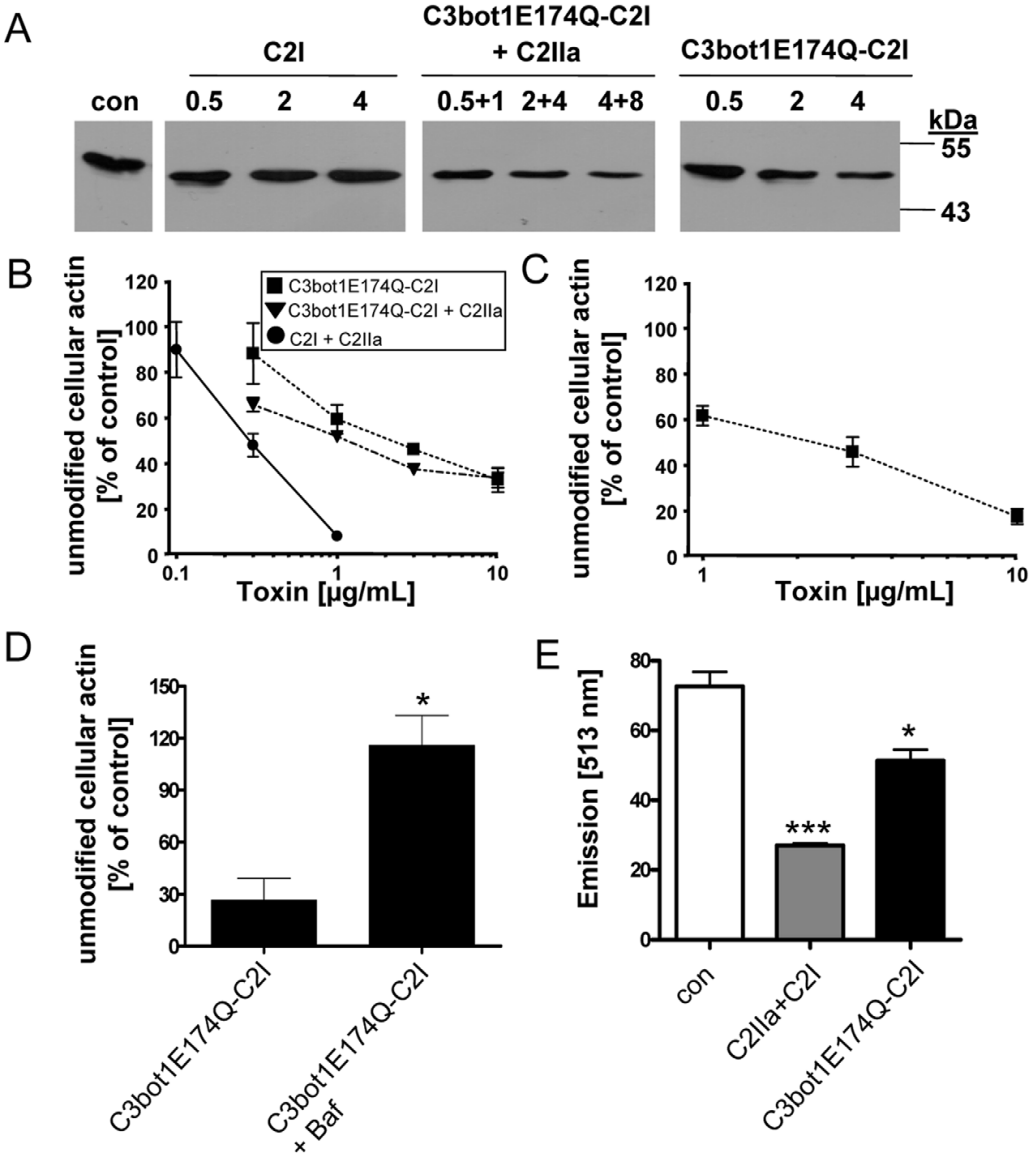
C3bot1E174Q-C2I ADP-ribosylates actin in the cytosol of intact J774A.1 and RAW264.7 macrophages. *A.* J774A.1 cells were incubated with C3bot1E174Q-C2I (0.5 µg/mL, 2 µg/mL, 4 µg/mL), C3bot1E174Q-C2I+C2IIa (0.5 µg/mL+1 µg/mL, 2 µg/mL+4 µg/mL, 4 µg/mL+8 µg/mL), C2I alone (0.5 µg/mL, 2 µg/mL, 4 µg/mL) or left untreated. Cells were lysed and lysates incubated for 30 min at 37°C with C2I (300 ng) and biotin-labelled NAD^+^ (10 µM) to ADP-ribosylate actin, which was not ADP-ribosylated by the toxins in the intact cells. Samples were subjected to SDS-PAGE, blotted and biotinylated (i.e. ADP-ribosylated) actin was detected with streptavidin-peroxidase. Comparable amounts of total protein in the lanes were confirmed by Ponceau S staining (shown in Figure S3). *B.* Concentration-dependent intoxication of J774A.1 cells with C3bot1E174Q-C2I, C3bot1E174Q-C2I+C2IIa or C2I+C2IIa. Cells were incubated for 6 h with 0.1, 0.3, 1, 3 or 10 µg/mL of the respective or left untreated for control. Subsequently, cells were lysed and further treated as described in A. Intensity of the bands showing ADP-ribosylated actin was determined by densitometry and is given as percentage of actin from untreated control cells (mean±S.D.; n = 3). Comparable protein loading was confirmed by Ponceau S staining (not shown). *C.* Concentration-dependent intoxication of RAW264.7 cells with C3bot1E174Q-C2I. Cells were incubated for 6 h with 0.1, 0.3, 1, 3 or 10 µg/mL C3bot1E174Q-C2I or left untreated for control. Cells were lysed and treated as described in B. Intensity of ADP-ribosylated actin was determined by densitometry and is given as percentage of actin from untreated control cells (mean±S.D.; n = 3). Comparable protein loading was confirmed by Ponceau S staining (not shown). *D*. Effect of bafilomycin A1 on intoxication of J774A.1 cells. Cells were pre-treated with 300 nM bafilomycin A1 (Baf) at 37°C and after 30 min C3bot1E174Q-C2I (3 µg/mL) or for control C2I (200 ng/mL)+C2IIa (400 ng/mL) was added to the medium. For control cells were left untreated. Cells were incubated for further 6 h, lysed and further treated as described in A. Intensity of the bands showing ADP-ribosylated actin was determined by densitometry and is given as percentage of actin from untreated control cells (mean±S.D.; n = 3; * = p≤0.5). *E.* F-actin staining of RAW.264.7 cells treated with C2 toxin or C3bot1E174Q-C2I. Cells seeded in a 96 well plate were treated for 24 h with either C2 toxin as a control (400 ng/mL C2IIa+200 ng/mL C2I) or with C3bot1E174Q-C2I (4 µg/mL) or left untreated. Cells were fixed, permeabilized and F-actin was stained with phalloidin-Alexa-591 and fluorescence detected at 513 nm with an ELISA reader (mean±S.D.; n = 3; * = p≤0.5, *** = p≤0.005).

To test whether C3bot1E174Q-C2I is taken up into the cytosol of cultured macrophages as an active enzyme, J774A.1 cells were incubated for 6 h with increasing amounts of C3bot1E174Q-C2I in the medium. For control, cells were incubated with C3bot1E174Q-C2I plus the separate C2IIa transport component that binds to the C2I moiety of C3bot1E174Q-C2I and delivers the fusion toxin into the cytosol, independent from the C3 portion. For further controls, cells were incubated with C2I alone, which should not enter the cells in the absence of C2IIa, and were left untreated (Fig. 3A, con). To analyze whether the toxins have ADP-ribosylated actin in the cytosol during the incubation period, cells were lysed and subjected to ADP-ribosylation *in vitro*. To this end, the lysates were incubated with biotin-NAD^+^ and fresh C2I as an enzyme. Actin from cells, which were either incubated without a toxin (Fig. 3A, con) or with C2I in the medium, was strongly ADP-ribosylated (i.e. biotin-labeled) by C2I during the *in vitro* reaction resulting in strong signals in the Western blot (Fig. 3A). This result clearly indicates that C2I alone is not taken up into the cytosol. In contrast, less biotin-labeled actin was detected when the cells were incubated with either C2IIa+C3bot1E174Q-C2I or with C3bot1E174Q-C2I alone. This indicates that in both cases some actin was already ADP-ribosylated in the living cells during incubation with the toxins. As shown in Fig. 3B, the amount of actin which was ADP-ribosylated in the intact cells correlated with the concentration of the toxin in the medium. Taken together, the results indicate that C3bot1E174Q-C2I was taken up into the cytosol of J774A.1 cells where the C2I portion of this fusion toxin ADP-ribosylated actin. This confirms the specific uptake of C3bot1E174Q-C2I and implies that the transport into the cytosol is mediated by its C3 moiety.

However, the C3bot1E174Q-C2I-catalyzed ADP-ribosylation of actin was more efficient when the fusion toxin was applied in combination with C2IIa. This might be due to the more efficient delivery of the fusion toxin via the C2IIa-specific uptake mechanism and/or to the fact that one portion of the applied C3bot1E174Q-C2I is delivered into the cytosol via CIIa and, in addition, another portion via the C3-specific uptake mechanism during the same time. In any case, the C3bot1E174Q-induced modification of actin in J774A.1 cells was not as strong as observed after treatment of cells with C2 toxin, implying that the uptake of C2 toxin into the cytosol was more efficient, independent whether C3bot1E174Q-C2I was applied to J774A.1 cells with or without C2IIa (Fig. 3B). When J774A.1 cells were treated for 30 min with bafilomycin A1, an inhibitor of endosomal acidification, prior to application of C3bot1E174Q-C2I less actin was ADP-ribosylated in the cytosol of these cells compared to cells treated with C3bot1E174Q-C2I in the absence of bafilomycin (Fig. 3D). This result strongly suggests that the uptake of C3bot1E174Q-C2I is inhibited by bafilomycin and implies that C3bot1E174Q-C2I translocates from acidified endosomal vesicles into the cytosol of macrophages as demonstrated for C3bot1 earlier [13].

Importantly, the uptake of C3bot1E174Q-C2I into the cytosol was not restricted to this macrophage line as demonstrated by the use of RAW264.7 cells (Fig. 3C and E as well as Fig. S1). Treatment of RAW264.7 cells with C3bot1E174Q-C2I resulted in a significantly decreased amount of F-actin after 24 h as determined by F-actin staining with fluorescent-labelled phalloidin (Fig. 3E). This indicates that the C3bot1E174Q-C2I-mediated ADP-ribosylation of actin resulted in depolymerization of F-actin, which is well known for C2 toxin. As described before for J774A.1 cells, no uptake of C2I alone was observed in RAW.264.7 cells (see Fig. S1), demonstrating the specific C3bot1E174Q-dependent transport in these cells.

C3bot1E174Q-C2I did not cause ADP-ribosylation of actin when it was administered to epithelial cell lines such as HeLa or African Green monkey kidney (Vero) cells (Fig. S2), implying that C3bot1E174Q-C2I was not delivered into the cytosol of these cells. In contrast, actin was ADP-ribosylated when C3bot1E174Q-C2I was delivered into the cytosol of Vero cells by the separate C2IIa transport component (Fig. S2A). This was confirmed by the toxin-induced changes in cell morphology. Due to the C2I activity in the cytosol, HeLa as well as Vero cells rounded up when C3bot1E174Q-C2I was introduced into the cytosol via C2IIa, but not, when the same amount of C3bot1E174Q-C2I was applied in the absence of C2IIa (Fig. S2B and C). Taken together, the results clearly demonstrate that C3bot1E174Q-C2I was enzymatic active when it was introduced into epithelial cells by an alternative mechanism (i.e. C2IIa-mediated delivery) but did not modify actin when it was applied alone to epithelial cells. In combination with earlier observations that C3 is selectively taken up into the cytosol of monocytes/macrophages, the results imply that the fusion toxin is not delivered into the cytosol of such cells.

### C3bot1E174Q-C2I but not C2I is Taken up into the Cytosol of Macrophages

**Figure 4:**
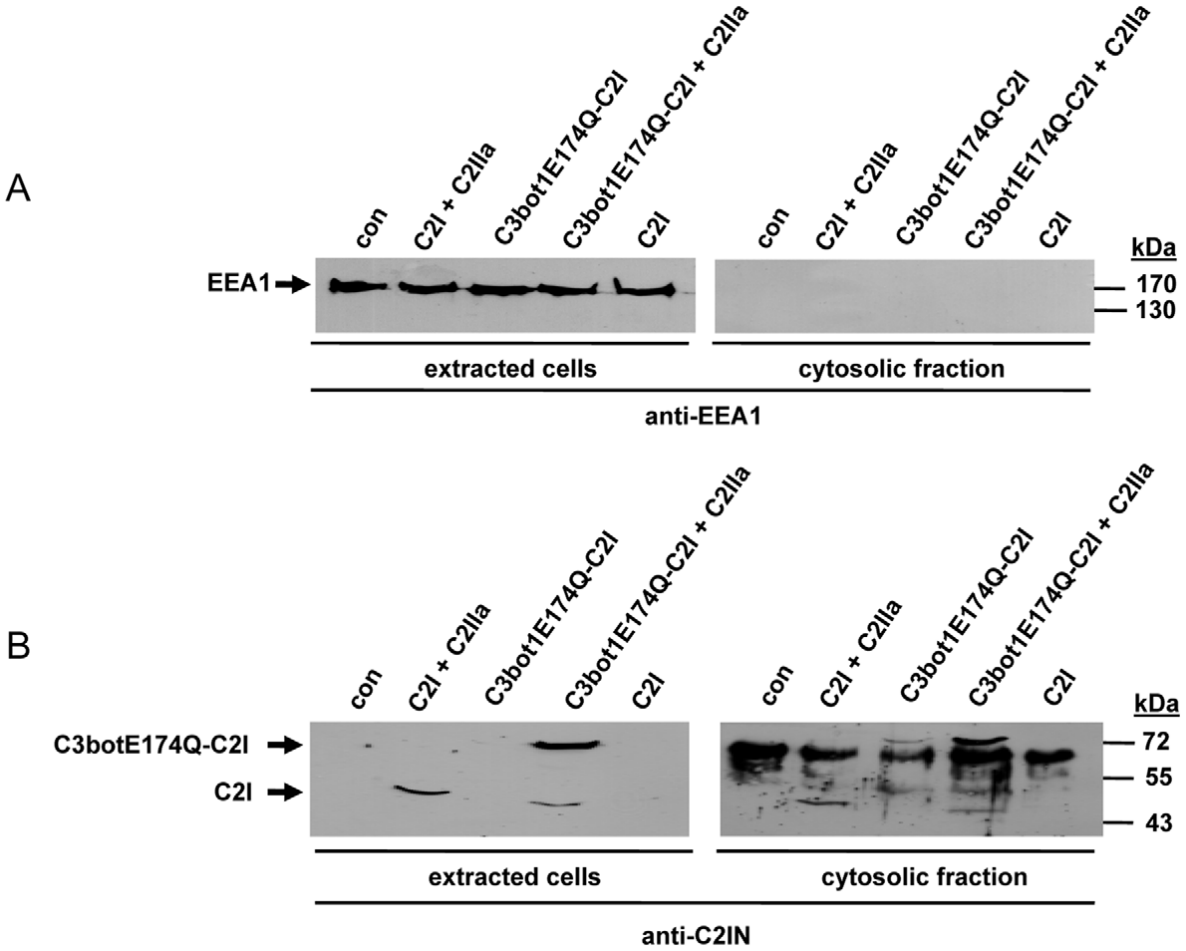
C3bot1E174Q-C2I is taken up into the cytosolic fraction of J774A.1 cells. *A.* J774A.1 cells were incubated for 6 h at 37°C with C3bot1E174Q-C2I (4 µg/mL). For control, cells were incubated for the same time with either C2I (1 µg/mL)+C2IIa (2 µg/mL) or C3bot1E174Q-C2I (4 µg/mL)+C2IIa (8 µg/mL). For further control cells were left untreated or were incubated with C2I alone (4 µg/mL). Thereafter, cells were washed, and incubated for 5 min at 4°C with an antibody against C2I (1∶2,000) to remove non-internalized C2I or C3bot1E174Q-C2I. After an additional washing step, cells were incubated with digitonin (20 µg/mL in PBS) to extract the cytosolic proteins. The cytosolic fraction as well as the remaining cells were subjected to SDS-PAGE and characterized by Western blotting. Comparable amounts of total protein in the lanes were confirmed by Ponceau S staining. An antibody against the endosomal protein EEA1 was used to confirm that the cytosolic fraction did not contain early endosomal vesicles. *B.* C2I and C3bot1E174Q-C2I were detected with an antibody against C2I.

J774A.1 cells were incubated with C3bot1E174Q-C2I and, subsequently the cytosolic fraction of the cells was prepared by digitonin extraction. The successful separation of the cytosolic fraction was confirmed by Western blot analysis with antibodies against the early endosomal antigen 1 (EEA-1) (Fig. 4A). Importantly, no EEA-1 was detectable in the cytosolic fraction, implying that there was no contamination of early endosomal vesicles which could contain internalized toxin. The cytosolic fractions as well as the extracted cells were then probed with an antibody against C2I to detect C3bot1E174Q-C2I. As shown in Fig. 4B, C3bot1E174Q-C2I was detected in the cytosolic fraction but not in the extracted cells, indicating that the internalized protein was transported into the cytosol. As observed before, the C2IIa-mediated delivery of C3bot1E174Q-C2I into the cytosol was more efficient compared to the C3bot1E174Q-mediated uptake of C3bot1E174Q-C2I. However, when C3bot1E174Q-C2I was applied to cells in combination with C2IIa, C3bot1E174Q-C2I was detected in the cytosolic fraction as well as in the extracted cells, implying that only a portion of the internalized C3bot1E174Q-C2I translocated into the cytosol. When the toxin is taken up via the C2IIa-dependent pathway (Fig. 4B, C3bot1E174Q-C2I+C2IIa), a profound portion of C3bot1E174Q-C2I remained in endosomal vesicles. A comparable result was obtained when C2I was taken up via C2IIa (Fig. 4B, C2I+C2IIa). As expected, C2I was neither detectable in the cytosolic fraction nor in the extracted cells when it was applied to cells without C2IIa (Fig. 4B). This is agreement with the established observation that C2I alone is not able to enter cells and a further strong hint that C3bot1E174Q-C2I is specifically taken up into J774A.1 cells via its C3- and not via its C2I-portion. Consistently, C3bot1E174Q-C2I was not taken up into the cytosol of Vero cells in the absence of C2IIa (data not shown).

**Figure 5:**
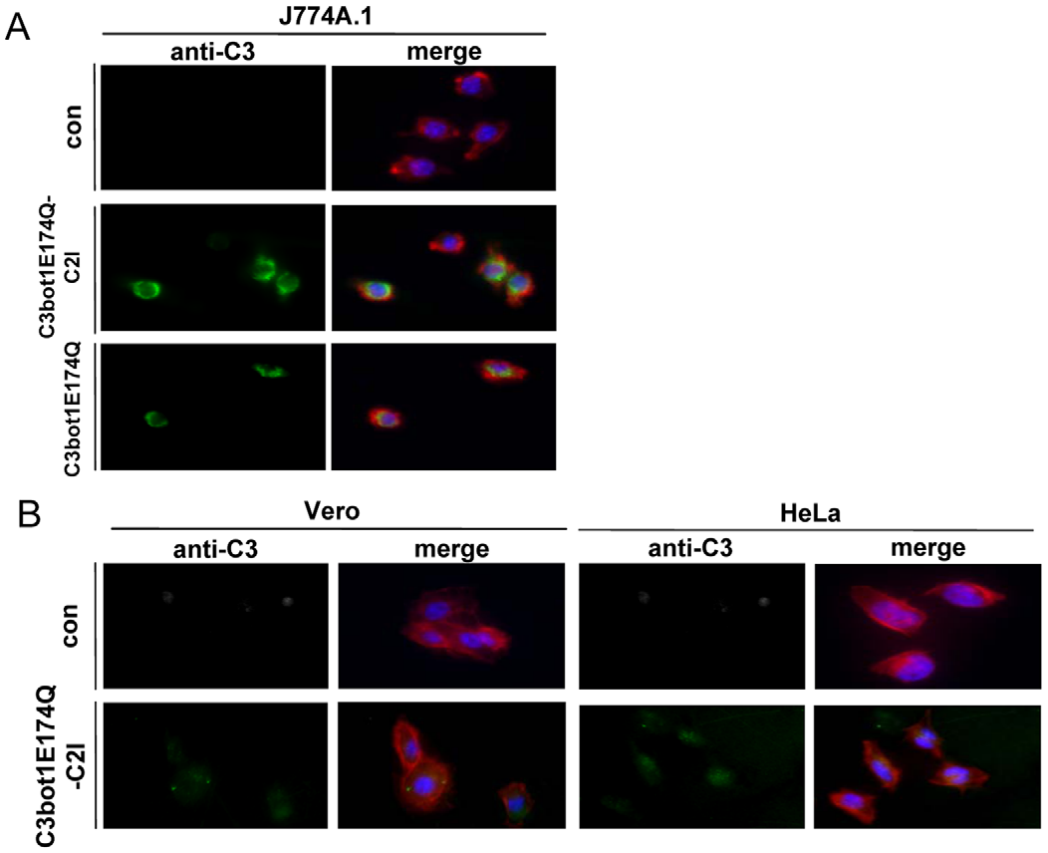
Immunofluorescence microscopy of C3bot1E174Q-C2I-treated J774A.1 and epithelial cells. *A.* J774A.1 cells grown on coverslips were incubated at 37°C with C3bot1E174Q-C2I (4 µg/mL), with C3bot1E174Q (4 µg/mL) or left untreated. *B.* Epithelial Vero and HeLa cells were incubated at 37°C with C3bot1E174Q-C2I (4 µg/mL) or left untreated. After 6 h, cells were fixed, permeabilized and stained with an antibody against C3bot and a secondary antibody coupled to Alexa 488 (green). The actin filaments were visualized using phalloidin-Alexa 594 (red) and nuclei were stained with Hoechst (blue). The cells were embedded in ProLong Gold antifade and analyzed by immunofluorescence microscopy.

**Figure 6:**
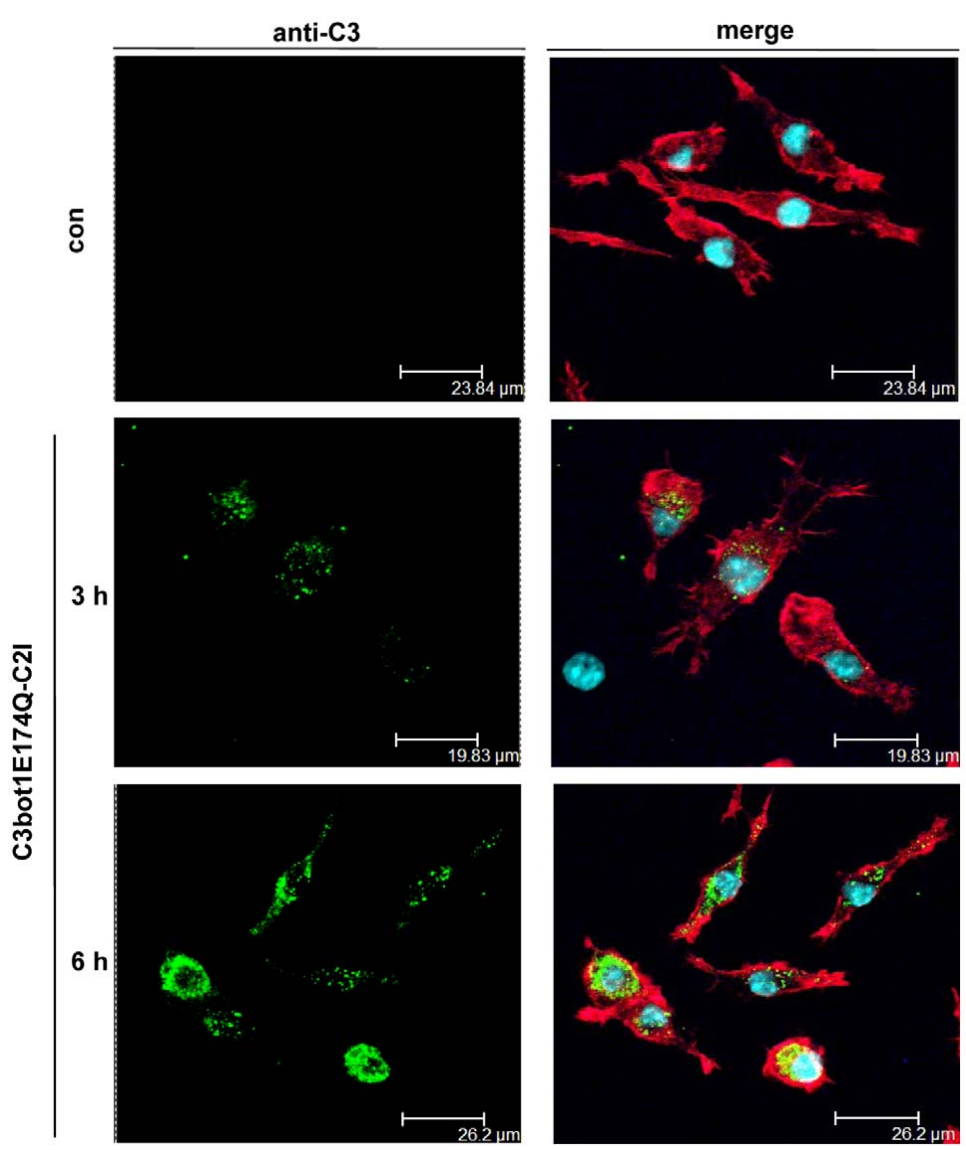
Confocal fluorescence microscopy of C3bot1E174Q-C2I-treated J774A.1 cells. J774A.1 cells grown on coverslips were incubated at 37°C with C3bot1E174Q-C2I (4 µg/mL) or left untreated as a negative control. After 3 h as well as 6 h cells were fixed, permeabilized and the toxin was stained with a primary antibody against C3bot and visualized using an Alexa 488-coupled antibody (green). The actin cytoskeleton was visualized using phalloidin-Alexa 594 (red). Slides were mounted with Fluoromount-G containing DAPI (blue) and analyzed by confocal microscopy.

The cellular uptake of C3bot1E174Q-C2I was also investigated by fluorescence microscopy (Fig. 5). In agreement with the findings described before, the results clearly revealed an uptake of C3bot1E174Q-C2I into macrophages (Fig. 5A) but not into epithelial cells, which corresponds to the cell-type selective uptake of C3bot1E174Q (Fig. 5B). Confocal fluorescence microscopy of C3bot1E174Q-C2I-treated J774A.1 cells revealed that the internalized C3bot1E174Q-C2I was mainly localized in the perinuclear region of J774A.1 cells after 3 h and more diffusely distributed after 6 h (Fig. 6).

### Uptake of C3bot1E174Q-C2I into Primary Macrophages

**Figure 7:**
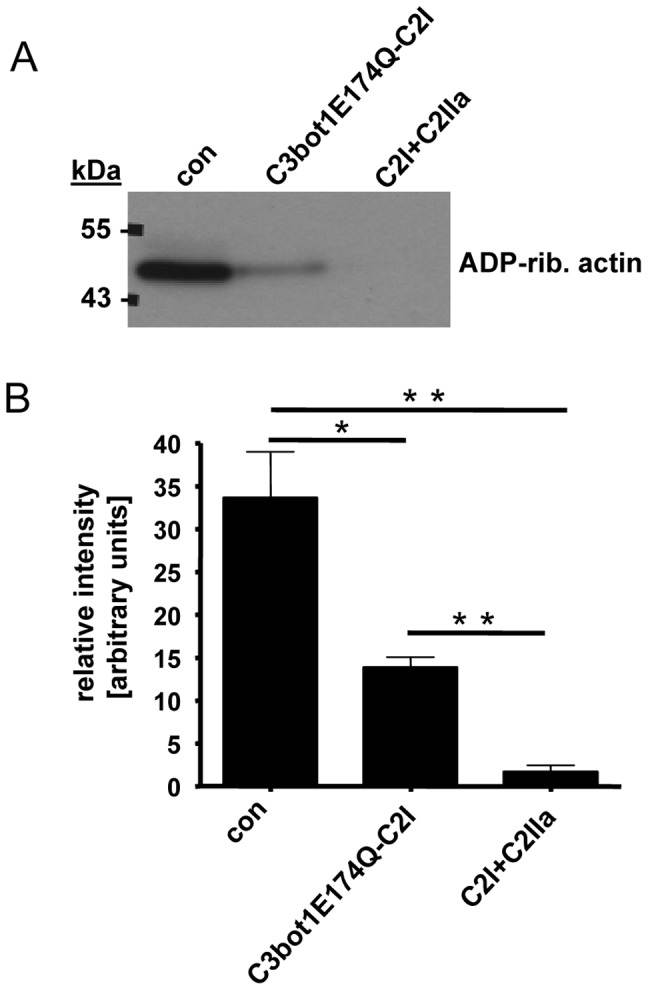
Effect of C3bot1E174Q-C2I on primary cultured macrophages. Monocytes from human blood were differentiated for 7 days. The resulting macrophages were treated for 6 h at 37°C with C3bot1E174Q-C2I (4 µg/mL) or for control with C2I+C2IIa (200+400 ng/mL) or left untreated. Cells were lysed and lysates incubated for 30 min at 37°C with C2I (300 ng) and biotin-labelled NAD^+^ (10 µM) to ADP-ribosylate unmodified actin. Comparable amounts of lysate protein were confirmed by SDS-PAGE and Coomassie staining (not shown). The biotinylated (i.e. ADP-ribosylated) actin was detected by Western blotting with streptavidin-peroxidase. *B.* Intensity of the bands showing ADP-ribosylated actin was determined by densitometry and is given as percentage of actin from untreated control cells (mean±S.D.; n = 3; * = p≤0.5, ** = p≤0.05). *B.* Comparable amounts of total protein in the lanes were confirmed by Ponceau S staining and anti-Hsp90 Western blotting. *B.* Morphology of the cells described in A after 6 h. *C.* HeLa cells were incubated with C3bot1E174Q-C2I (4 µg/mL)+C2IIa (8 µg/mL) or with C3bot1E174Q-C2I alone (4 µg/mL). For control cells were left untreated or were incubated with C2I alone (4 µg/mL). After 6 h of incubation at 37°C all cells were washed, incubated with an antibody against C2I for 5 min at 4°C to remove non-internalized C2I and C2I fusions, washed again and pictures were taken.

Finally, the uptake of C3bot1E174Q-C2I into primary cultured human macrophages was investigated. Monocytes from blood from human donors were *in vitro* differentiated and incubated for 6 h with C3bot1E174Q-C2I or with C2I+C2IIa. For control cells were left untreated. Subsequently the ADP-ribosylation status of actin from these cells was analyzed by sequential ADP-ribosylation. As shown in Fig. 7, weaker signals for *in vitro* ADP-ribosylated actin were obtained when the cells were treated with one of the toxins prior to their lysis, indicating that some actin was already ADP-ribosylated in the intact cells during incubation with the toxins. As observed for the J774A.1 and RAW264.7 cells, C2 toxin was more efficient then the C3bot1E174Q-C2I fusion toxin. However, this result clearly demonstrates that C3bot1E174Q-C2I is taken up into the cytosol of primary cultured human macrophages.

Taken all together, we have cloned, purified and characterized a recombinant fusion toxin which exploits the monocyte/macrophage-selective uptake mechanism of a clostridial C3bot protein to deliver a foreign enzyme into the cytosol of macrophage. Importantly, an 6 h treatment with 4 µg/mL C3bot1E174Q did not result in an increased release of tumor necrosis factor (TNF) alpha from J774A.1 cells as measured by ELISA (data not shown), indicating that the transport moiety C3botE174Q did not interfere with macrophage activation under such conditions.

## Discussion

Here, we have demonstrated that enzymatic inactive C3bot1 protein can be used as a carrier for selective delivery of recombinant fusion toxins into the cytosol of macrophages including primary cultured human macrophages. Therefore, we constructed and characterized the recombinant C3bot1E174Q-C2I protein and we demonstrated that this fusion toxin ADP-ribosylates actin in living macrophages but not in epithelial cells. No uptake of this fusion toxin into epithelial cells was observed under the conditions used in this study while it was efficiently delivered into the cytosol of macrophages within 3–6 h of incubation. In summary, the results imply that the cell-type selective uptake of this fusion toxin is mediated by its C3bot1E174Q moiety because C3bot1E174Q but not C2I is delivered into the cytosol of macrophages. This is in agreement with our earlier observations that clostridial C3 proteins selectively enter monocytes/macrophages when relatively low concentrations of C3 (<1 µg/mL) were applied for relatively short incubation periods (<3 h) [13]. In contrast, fibroblasts (CHO-K1) and epithelial cells (HeLa, Vero) did not respond to C3-treatment, even when these cells were challenged with 10 µg/mL of C3 for 24 h [13]. Only when higher concentrations of C3 were applied to these cell types for more than 24 h, some cells showed the characteristic “C3-morphology”, most likely due to a non-specific uptake of C3. The more than 10-fold higher sensitivity of monocytes (e.g. HL-60) and macrophages (e.g. RAW264.7 and J774A.1) towards C3 compared to epithelial cells and fibroblasts should allow for titration of recombinant C3-based fusion toxins and, thereby a selective targeting of monocytes/macrophages in tissues and probably whole organisms.

Recently, we have demonstrated that C3bot1E174Q delivers streptavidin into the cytosol of monocytes/macrophages after chemical crosslinking of streptavidin to the C3 protein [40]. This bioconjugate was able to transport biotinylated molecules including small fluorescent compounds as well as proteins into such cells. However, the cell-type selectivity of C3 was lost to a certain extend as the bioconjugate was also taken up by epithelial cells and fibroblasts [40]. This might be due to the streptavidin moiety, as this effect was also reported for other streptavidin conjugates [41].

The uptake mechanism of C3bot1 into the cytosol macrophages is not completely understood and no receptor for C3 on the surface of macrophages identified so far. Moreover, no receptor-binding domain was identified in C3 proteins. However, there is evidence that C3 is internalized by receptor-mediated endocytosis and at least a portion of the internalized C3 protein translocates from acidified endosomal vesicles into the cytosol [13]. The mechanism how C3 translocates across intracellular membranes also requires further investigation. Interstingly, C3bot1 as well as the closely related C3lim from *C. limosum* require acidic conditions to interact with lipid bilayer membranes *in vitro* and to translocate across cell membranes in living cells [13]. This pH-dependent membrane translocation was observed for a variety of bacterial AB-type toxins including the binary C2 toxin from *C. botulinum* [27], which form trans-membrane pores in endosomal membranes [28], [29] and translocate as (at least partially) unfolded proteins across the membranes from endosomes into the cytosol [31]. C2 toxin and some other toxins exploit the activities of host cell chaperones and folding helper enzymes for translocation and/or refolding [32], [33], [34]. It remains to be elucidated, whether C3 proteins also require unfolding to translocate and, if so, how they become refolded in the host cell cytosol. Moreover, no translocation domain was found in the clostridial C3 proteins so far. The intracellular membrane translocation process might be a limiting step for the delivery of C3-fusion toxins into the cytosol and thus could explain the finding that not the entire portion of the internalized C3bot1E174Q-C2I protein finally reached the cytosol.

In our hands, C3bot1E174Q did not exhibit adverse effects in terms of cell morphology, viability, proliferation or activation of cultured J774A.1 macrophages. Therefore, the C3bot1E174Q-induced cell cycle arrest which was recently reported for the murine hippocampal cell line HT22 [42] might be a cell-type specific effect.

In summary, we demonstrated that enzymatic inactive C3bot1E174Q protein serves as a protein delivery system which allows fast, selective and specific transport of enzymes into the cytosol of living macrophages including primary human macrophages differentiated from blood monocytes. In Recombinant C3-fusion toxins can represent attractive and simple tools in experimental monocyte/macrophage pharmacology as well as potential candidates for the development of novel therapeutic approaches against monocyte/macrophage-associated diseases.

## Materials and Methods

### Materials and Reagents

Cell culture media (DMEM and MEM), fetal calf serum and penicillin-streptomycin were from Invitrogen (Karlsruhe, Germany). Cell culture materials were from TPP (Trasadingen, Switzerland). Complete® protease inhibitor and streptavidin-peroxidase were from Roche (Mannheim, Germany). Page Ruler pre-stained Protein ladder® was from Fermentas (St. Leon Rot, Germany). Biotinylated NAD^+^ was from R&D Systems GmbH (Wiesbaden-Nordenstadt, Germany). Anti-C2IN antibody and anti-C3bot1E174Q antibody were raised in rabbits from Pineda (Berlin, Germany). Antibody against Hsp90 was from Santa Cruz Biotechnology (Heidelberg, Germany). Enhanced chemiluminescence (ECL) system was obtained from Millipore (Schwalbach, Germany). The nitrocellulose blotting membrane was from Whatman® (Dassel, Germany). Oligonucleotides were purchased from Thermo-Scientific (Ulm, Germany), the DNA molecular weight marker (Smart Ladder®) from Eurogentec (Köln, Germany), the restriction enzymes and the T4 ligase from New England Biolabs (Frankfurt, Germany). Thrombin was from Amersham Biosciences Europe GmbH (Freiburg, Germany). Digitonin was obtained from Sigma-Aldrich (München, Germany). Alexa-488 coupled antibody and phalloidin-Alexa 594 coupled conjugate were purchased from Invitrogen (Karlsruhe, Germany). The enzyme-linked immunosorbent assay (ELISA), detecting tumor necrosis factor alpha (TNFα), was obtained from Becton, Dickinson and Company (Heidelberg, Germany). The human monocyte enrichment kit was from Stemcell Technologies (Köln, Germany). Human M-CSF was purchased from Peprotech (Hamburg, Germany).

### Construction of C3bot1E174Q-C2I

The C3bot1E174Q gene (633 bp, kindly provided by Dr. K. Aktories, Freiburg, Germany) was amplified by PCR with 400 ng of chromosomal DNA in a total volume of 50 µl with 1 U of Pwo-DNA-polymerase in a reaction mixture including deoxynucleoside triphosphates (200 µM each) and 0.3 µM of the primers C3 forward (5′-GGTGGTGGATCCGCTTATTCAAATACTTAC-3′), containing a Bam*HI* site and C3 reverse (5′-GAATTCGGCCTGAGATCTTTTAGGATTGATAGC-3′), containing a Eco*RI* and a *Bgl*II site. Amplification was done by 30 cycles of denaturing at 94°C for 15 s, primer annealing at 60°C for 30 s and elongation at 68°C for 100 s. The C2I gene (1,296 bp) from *C. botulinum* was amplified by PCR with 100 ng of chromosomal DNA in a total volume of 50 µl with 1 U of Pwo-DNA-polymerase in a reaction mixture including deoxynucleoside triphosphates (200 µM each) and 0.3 µM of the primers C2I forward (5′-CCCGGGAGATCTCCAATAATAAAAGAACCC-3′), containing a Bgl*II* site and C2I reverse (5′-TGATCACGCCGCTCCCTAAATCTCTTTATTTTGTATACC-3′), containing a *Bcl*I site. Amplification was done by 30 cycles of denaturing at 94°C for 60 s, primer annealing at 50°C for 60 s and elongation at 68°C for 120 s. Each PCR product was ligated in the pCR-Blunt vector using the Zero Blunt PCR cloning kit (Invitrogen, Karlsruhe, Germany). The constructs pCRblunt-C3bot1E174Q and pCRblunt-C2I were sequenced by using the sequencing primers M13 forward (5′-TGTAAAACGACGGCCAGT-3′) and M13 reverse (5′-CAGGAAACAGCTATGACCA-3′). For expression experiments the isolated C3bot1E174Q gene was digested with *Bam*HI/*Eco*RI and ligated with a *Bam*HI/*Eco*RI digested pGEX2T vector. The plasmid C3bot1E174Q-C2I was constructed by digesting the plasmid pCRblunt-C2I with *Bgl*II*/Eco*RI followed by a ligation with *Bgl*II*/Eco*RI digested pGEX2T-C3bot1E174Q. The construct pGEX2T-C3bot1E174Q-C2I was sequenced by using the sequencing primers pGEX5 (5′-CTGGCAAGCCACGTTTGG-3′), pGEX3 (5′-GGAGCTGCATGTGTCAGAG-3′) and C3–5′ (5′-AACACTCTTAATTCAAATGG -3′).

### Expression and Purification of C3bot1E174Q-C2I

C3bot1E174Q-C2I was over-expressed as GST-tagged fusion protein in *E.coli* (BL21) and purified by affinity chromatography. In brief, *E.coli* (BL21) harboring plasmid pGEX2T-C3bot1E174Q-C2I was grown at 37°C in Luria-Bertani (LB) medium supplemented with 100 µg/mL ampicillin to an optical density of 0.6–0.8. To induce protein expression, isopropyl-ß-D-thiogalactopyranoside (IPTG) was added to a final concentration of 0.2 mM and the cultures incubated at 29°C for 5 h. The bacteria were harvested by centrifugation for 10 min at 4°C at 5,000 rpm, resuspended in lysis buffer (10 mM NaCl, 20 mM Tris, pH 7.4), containing 0.1% Triton X-100, 1% PMFS and 1∶50 Complete® protease inhibitor and disrupted by sonification. Cellular debris was sedimented for 15 min at 4°C at 12,000 rpm and the clear supernatant was incubated with glutathione-agarose beads (Macherey-Nagel, Düren, Germany) for 1 h at room temperature. The suspension was centrifuged for 3 min at room temperature at 2,200 rpm, the beads were washed 2 times with wash buffer (150 mM NaCl, 20 mM Tris, pH 7.4) and one time with PBS and finally the immobilized protein was incubated with thrombin (4 NIH units/L culture) for 1 h at room temperature to remove the GST-tag. The supernatant containing C3bot1E174Q-C2I was obtained by centrifugation for 30 s at 10,000 g at 4°C and for the elimination of thrombin the supernatant was incubated with benzamidine beads (GE Healthcare, München, Germany) for 10 min at room temperature. The fusion protein (in PBS) was collected and for protein stability 1∶50 Complete® protease inhibitor was added. The identity of the fusion toxin was confirmed by immunoblot analysis with antibodies against C3bot and C2I.

### Cell Culture and Intoxication Assays

Cells J774A.1 macrophage-like cells (from DSMZ, Braunschweig, Germany) and RAW264.7 cells (from LGC Standards GmbH, Wesel, Germany) were cultivated at 37°C and 5% CO_2_ in DMEM medium, containing 10% heat-inactivated fetal calf serum and penicillin-streptomycin (1∶100). Cells were routinely scrapped off and reseeded three times per week. Vero and HeLa cells (from DSMZ, Braunschweig, Germany) were cultivated at 37°C and 5% CO_2_ in MEM medium, containing 10% heat-inactivated fetal calf serum, 1 mM sodium-pyruvate, 2 mM L glutamine, 0.1 mM non-essential amino acids and penicillin-streptomycin (1∶100). Cells were trypsinized and reseeded three times per week. For intoxication experiments, cells were seeded in 12-well culture dishes and incubated with the respective toxin in the medium at 37°C and 5% CO_2_. For control, cells were incubated without toxin. After 6 h of incubation the cells were washed, incubated with an antibody against C2I (1∶2,000) for 5 min (HeLa cells) or 15 min (Vero cells) at 4°C and washed again. To analyze the morphological changes caused by the toxins, the cells were visualized by using a Zeiss Axiovert 40CFI microscope (Oberkochen, Germany) with a Jenoptik progress C10 CCD camera (Jena, Germany).

### Preparation, Differentiation and Intoxication of Primary Macrophages

Monocytes were prepared from buffy-coats from human donors by using the human monocyte enrichment kit from Stemcell Technologies (Köln, Germany) according to the manufacturer’s instructions. Cells were seeded in 12-well plates at a density of 1×10^6^ cells per plate. Monocytes were differentiated for 7 days by enriching medium (DMEM, 10% heat inactivated FCS and penicillin-streptomycin, 1∶100) containing recombinant human M-CSF (macrophage colony stimulating factor, 50 ng/ml). The medium was changed every second day. After seven days the differentiated monocytes were treated in serum-free medium with C2I+C2IIa (200+400 ng/mL), C3bot1E174Q-C2I (4 µg/mL) or without toxin for control. After 6 h cells were lysed and subjected to sequential ADP-ribosylation of actin to monitor uptake of the toxins into the cytosol.

### Measurement of F-actin

RAW.264.7 cells in a 96 well plate were treated for 24 h with C2 toxin or C3bot1E174Q-C2I fusion toxin or left untreated for control. Subsequently, cells were fixed for 30 min with 4% paraformaldehyde and permeabilized with Triton-X100. Finally, F-actin was stained with phalloidin-Alexa-591 and after washing the fluorescence was detected at 513 nm with an Infinite M1000 ELISA plate reader from Tecan Group Ltd. (Crailsheim, Germany).

### SDS-PAGE and Immunoblot Analysis

For immunoblot analysis, equal amounts of protein were subjected to SDS-PAGE according to the method of Laemmli [38]. Subsequently, the proteins were transferred to a nitrocellulose membrane and transfer of comparable protein amounts was confirmed by Ponceau S staining. The membrane was then blocked for 30 min with 5% dry milk powder in PBS containing 0.1% Tween-20 (PBS-T). For the detection of the biotin-labelled actin the samples were probed with streptavidin-peroxidase. Subsequently, the membrane was washed and proteins visualized using a chemiluminescence (ECL) system according to the manufacturer’s instructions. For the detection of the fusion toxin C3bot1E174Q-C2I, the samples were probed with a rabbit anti-C2IN antibody or a rabbit anti-C3bot1E174Q antibody. After washing with PBS-T, the membrane was incubated for 1 h with an anti-rabbit antibody coupled to horseradish-peroxidase (Santa Cruz Biotechnology, Heidelberg, Germany). The membrane was washed and the proteins visualized in a subsequent chemiluminescence reaction.

### ADP-ribosylation of Actin by C3bot1E174Q-C2I in a Cell-free System

J774A.1 cell lysate (20 µg of protein) was incubated for 10 min at 37°C together with different concentrations of C3bot1E174Q-C2I and C2I respectively and 10 µM biotin-labelled NAD^+^. Samples were subjected to SDS-PAGE and blotted onto a nitrocellulose membrane. ADP-ribosylated, i.e. biotin-labelled, actin was detected with streptavidin-peroxidase.

### Sequential ADP-ribosylation of Actin in Lysates from Toxin-treated Cells

For ADP-ribosylation of actin in a cell-free system, 5 µg (Raw264.7 cells) or 10 µg (J774A.1 cells) of whole cell lysate protein were incubated for 30 min at 37°C in a buffer containing 20 mM Tris-HCl (pH 7.5), 1 mM EDTA, 1 mM DTT, 5 mM MgCl_2_, complete® protease inhibitor, together with biotin-labelled NAD^+^ (10 µM) and 300 ng of C2I protein. The reaction was stopped with 5 × SDS-sample buffer (625 mM Tris/HCl pH 6.8, 20% SDS, 8.5% glycerol, 0.2% bromphenol blue, 100 mM DTT) and heating of the samples for 10 min at 95°C. Subsequently the samples were subjected to SDS-PAGE and transferred to a nitrocellulose membrane. The biotin-labelled ADP-ribosylated actin was detected with peroxidase-coupled streptavidin and a subsequent chemiluminescence reaction.

### Detection of C3bot1E174Q-C2I in Cytosolic Fractions of J774A.1 Cells

The cytosolic fraction from cultured cells was obtained by treatment of cells with digitonin as described earlier [33]. The cells were grown in 12-well plates and incubated with the respective toxin for 6 h at 37°C. For a control, cells were incubated without toxin. To remove unbound toxin from the surface, the cells were washed twice with ice-cold PBS, incubated with an antibody against C2I (1∶2,000) for 5 min at 4°C and washed again. Subsequently, the cells were incubated for 5 min at 25°C in the presence of digitonin (20 µg/mL in PBS) to permeabilize the cell membrane and for an additional 25 min at 4°C to allow extraction of the cytosolic proteins, including C3bot1E174Q-C2I. The supernatant was collected and the remaining cells were scrapped off. Equal amounts of protein were subjected to SDS-PAGE and C3bot1E174Q-C2I was detected with an antibody against C2I using the ECL system. The absence of early endosomes in the cytosolic fraction was confirmed in an immunoblot with an antibody raised against EEA1 (Acris, Herford, Germany), a marker protein for early endosomes [39].

### Immunofluorescence Microscopy

For immunocytochemical analysis cells were seeded on sterile coverslips and incubated in 12-well culture dishes overnight. Then cells were incubated with the respective toxin for 6 h at 37°C or left untreated for control. Next, cells were fixed with 4% paraformaldehyde (PFA), permeabilized with 0.4% Triton X-100 and blocked with 5% dry milk powder in PBS-T. C3bot1E174Q-C2I and C3bot1E174Q were stained with a primary antibody against C3bot (1∶3,000 in 5% dry milk in PBS-T) and visualized using an Alexa 488-coupled antibody (1∶400 in 5% dry milk in PBS-T). After several washing steps in PBS the actin cytoskeleton was labeled with phalloidin-Alexa 594 (1∶200 in PBS) and nuclei were counterstained with Hoechst 33342 (1∶10,000 in 4% PFA). Finally, the cells were embedded in ProLong Gold antifade (Invitrogen, Karlsruhe, Germany) and analyzed by immunofluorescence microscopy using a Zeiss Axio observer Z1 microscope (Oberkochen, Germany). Images were collected using Metamorph software (MDS Analytical Technologies, Toronto, Canada), processed and merged with *ImageJ* software (NIH, Bethesda, USA).

### Confocal Laser Scanning Microscopy

To analyze the uptake of C3bot1E174Q-C2I, J774A.1 cells were seeded on coverslips and incubated in 12-well dishes overnight. Then cells were incubated with C3bot1E174Q-C2I for 3 or 6 h at 37°C. For control, cells were incubated without toxin. Next, cells were fixed with 4% PFA, permeabilized with 0.4% Triton X-100 and blocked with 5% dry milk in PBS-T. C3bot1E174Q-C2I was stained with a primary antibody against C3bot (1∶3,000 in 5% dry milk in PBS-T) and visualized using an Alexa 488-coupled antibody (1∶400 in 5% dry milk in PBS-T). After several washing steps in PBS the actin cytoskeleton was labeled with phalloidin-Alexa 594 (1∶150 in PBS). Finally, slides were mounted with Fluoromount-G (Southern Biotechnology, Birmingham, USA) containing DAPI. Images were obtained using a confocal microscope (Leica TCS SP2 equipment, objective lense; HeX PL APO, 40×/1.25–0.75) and LCS software (Leica Microsystems CMS GmbH, Mannheim, Germany). Subsequently images were processed with *ImageJ* software (NIH, Bethesda, USA).

### Measurement of Tumor Necrosis Factor (TNF) Alpha Released from Cultured Macrophages

J774A.1 cells grown for 2 days in 12-well plates were treated for 6 h in serum-free medium with C3bot1E174Q-C2I (4 µg/mL) or left untreated for control. Subsequently, 500 µL of the medium were removed, centrifuged at 8.000 rpm for 5 min and analyzed by enzyme linked immunosorbent assay (ELISA) for the amount of tumor necrosis factor alpha (TNFα) according to manufacturer’s instructions. For positive control, cells were incubated for 6 h with *E.coli* BL21 lysate to trigger LPS-induced macrophage activation and release of TNFα into the medium.

### Reproducibility of the Experiments and Statistics

All experiments were performed independently at least 2 times. Results from representative experiments are shown in the figures. In each individual immunoblot panel shown in the figures, the protein bands were originally detected on the same membrane and cut out and recombined for presentation in the figures.

## Supporting Information

Figure S1 **C3bot1E174Q-C2I ADP-ribosylates actin in the cytosol of intact RAW264.7 macrophages.** Raw264.7 cells were incubated for 6 h with C3bot1E174Q-C2I (0.5 µg/mL, 2 µg/mL), C3bot1E174Q-C2I+C2IIa (0.1 µg/mL+0.2 µg/mL, 0.4 µg/mL+0.8 µg/mL), C2I alone (2 µg/mL) or left untreated for control. *A.* Cells were lysed and lysates incubated for 30 min at 37°C with C2I (300 ng) and biotin-labelled NAD^+^ (10 µM) to ADP-ribosylate actin, which was not ADP-ribosylated by the toxins in the intact cells. Samples were subjected to SDS-PAGE, blotted and biotinylated (i.e. ADP-ribosylated) actin was detected with streptavidin-peroxidase. *B.* Comparable amounts of total protein in the lanes were confirmed by Ponceau S staining and anti-Hsp90 Western blotting.
(TIF)

Figure S2 **C3bot1E174Q-C2I has no effect on epithelial cells.** *A.* ADP-ribosylation status of actin. Vero cells were incubated with C2I (0.2 µg/mL)+C2IIa (0.4 µg/mL), C3bot1E174Q-C2I (2 µg/mL)+C2IIa (4 µg/mL) or with C3bot1E174Q-C2I alone (2 µg/mL). For control cells were left untreated. After 6 h of incubation at 37°C all cells were washed, incubated with an antibody against C2I (1∶2,000) for 15 min at 4°C to remove non-internalized C2I and C2I fusions, washed again and lysed. Lysates were incubated for 30 min at 37°C with C2I (300 ng) and biotin-labelled NAD^+^ (10 µM) to ADP-ribosylate actin, which was not ADP-ribosylated by the toxins in the intact cells. Samples were subjected to SDS-PAGE, blotted and biotinylated (i.e. ADP-ribosylated) actin was detected with streptavidin-peroxidase. Comparable amounts of total protein in the lanes were confirmed by Ponceau S staining and Western blot analysis of Hsp90. *B.* Morphology of the cells described in A after 6 h. *C.* HeLa cells were incubated with C3bot1E174Q-C2I (4 µg/mL)+C2IIa (8 µg/mL) or with C3bot1E174Q-C2I alone (4 µg/mL). For control cells were left untreated or were incubated with C2I alone (4 µg/mL). After 6 h of incubation at 37°C all cells were washed, incubated with an antibody against C2I for 5 min at 4°C to remove non-internalized C2I and C2I fusions, washed again and pictures were taken.
(TIF)

Figure S3 **Comparable protein loading in the experiment shown in** **Fig. 3A** **was confirmed by Ponceau S staining of the blot membrane.**
(TIF)
