# Supplementary figures and images for: A Recombinant Fusion Toxin Based on Enzymatic Inactive C3bot1 Selectively Targets Macrophages
Source: PLoS One. 2013 Jan 21;8(1):e54517. doi: 10.1371/journal.pone.0054517 (PMC3549961; doi:10.1371/journal.pone.0054517)

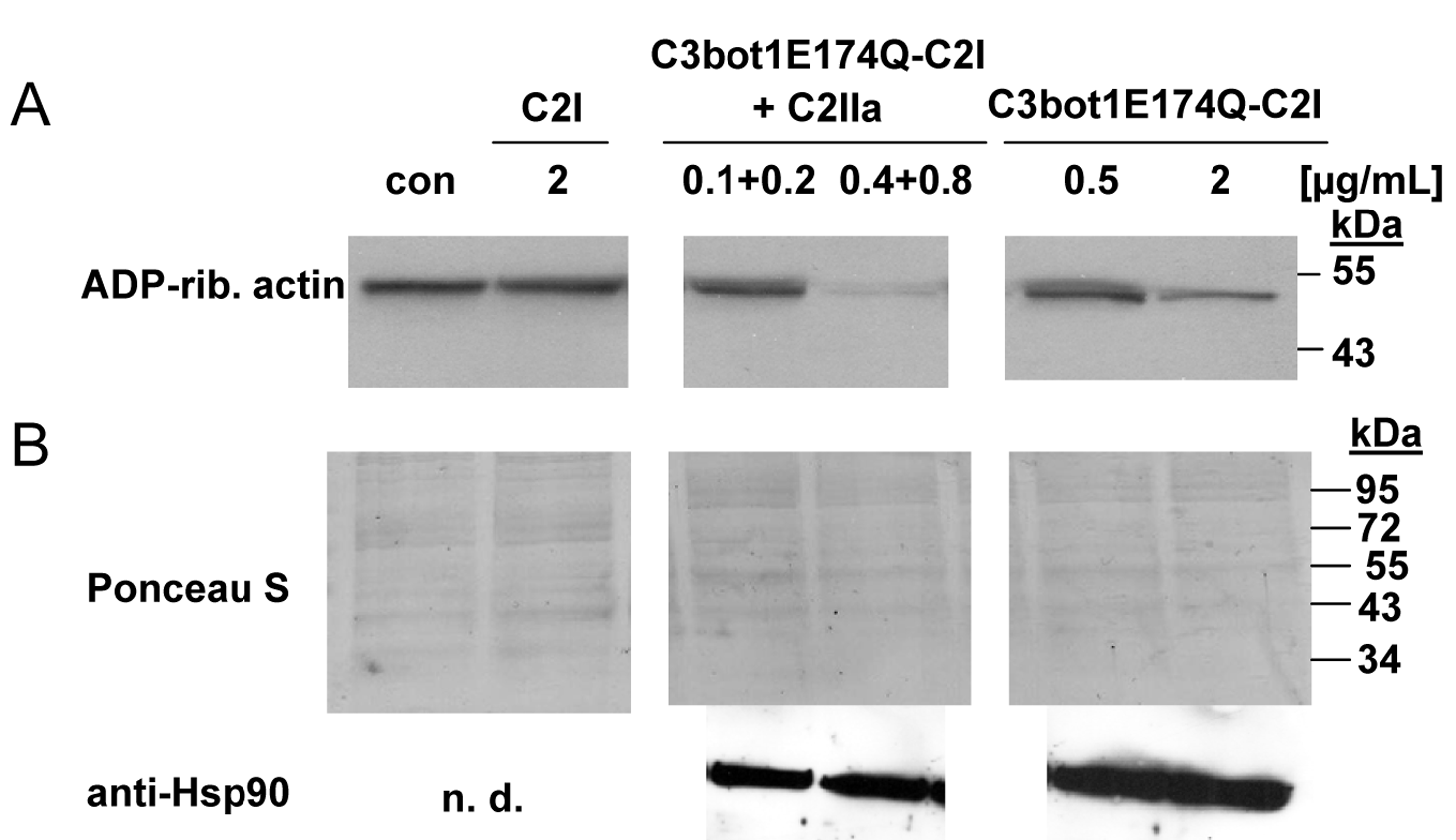

Supplement: Figure S1 — C3bot1E174Q-C2I ADP-ribosylates actin in the cytosol of intact RAW264.7 macrophages. Raw264.7 cells were incubated for 6 h with C3bot1E174Q-C2I (0.5 µg/mL, 2 µg/mL), C3bot1E174Q-C2I+C2IIa (0.1 µg/mL+0.2 µg/mL, 0.4 µg/mL+0.8 µg/mL), C2I alone (2 µg/mL) or left untreated for control. A. Cells were lysed and lysates incubated for 30 min at 37°C with C2I (300 ng) and biotin-labelled NAD+ (10 µM) to ADP-ribosylate actin, which was not ADP-ribosylated by the toxins in the intact cells. Samples were subjected to SDS-PAGE, blotted and biotinylated (i.e. ADP-ribosylated) actin was detected with streptavidin-peroxidase. B. Comparable amounts of total protein in the lanes were confirmed by Ponceau S staining and anti-Hsp90 Western blotting. (TIF) [file pone.0054517.s001.tif]

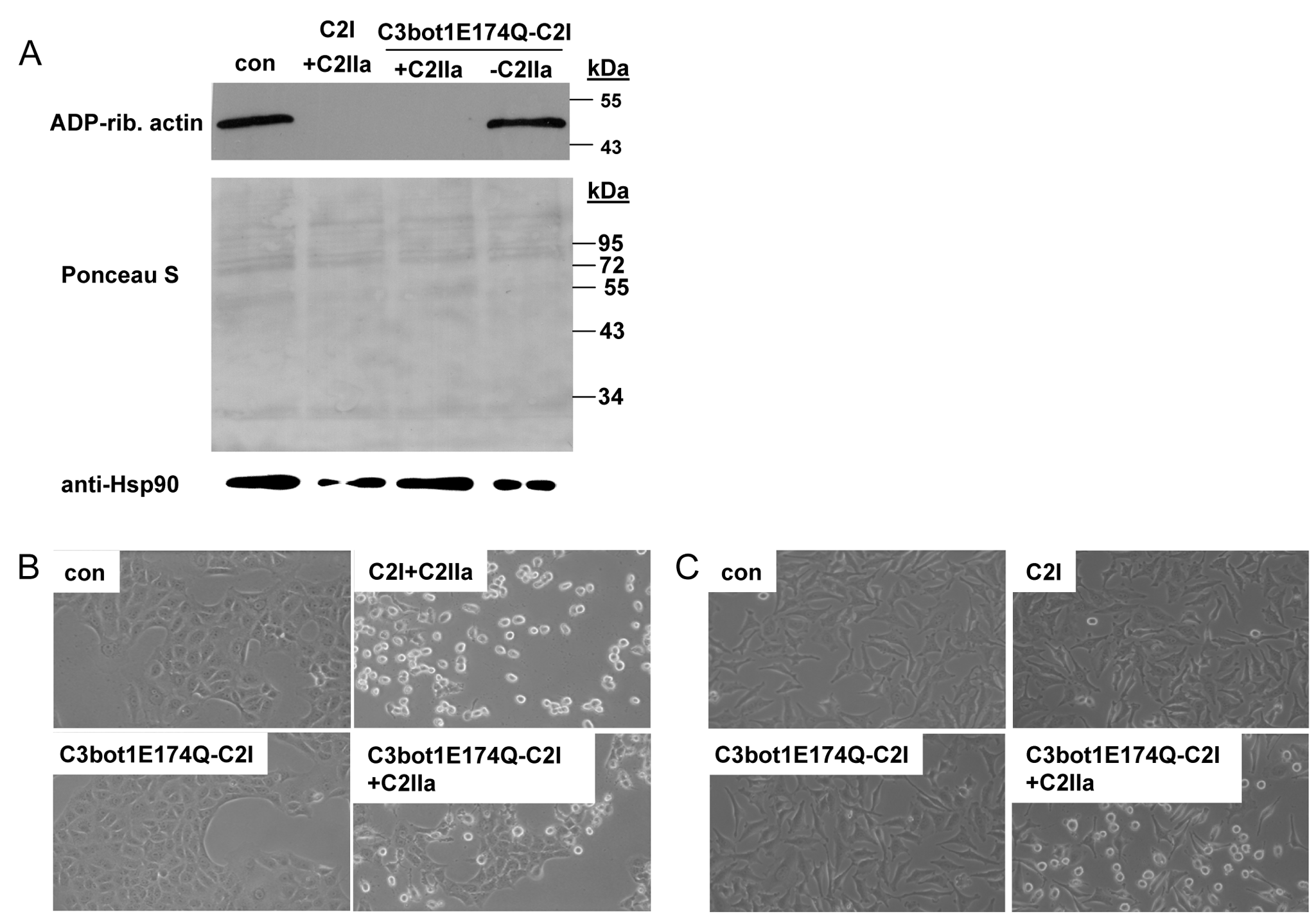

Supplement: Figure S2 — C3bot1E174Q-C2I has no effect on epithelial cells. A. ADP-ribosylation status of actin. Vero cells were incubated with C2I (0.2 µg/mL)+C2IIa (0.4 µg/mL), C3bot1E174Q-C2I (2 µg/mL)+C2IIa (4 µg/mL) or with C3bot1E174Q-C2I alone (2 µg/mL). For control cells were left untreated. After 6 h of incubation at 37°C all cells were washed, incubated with an antibody against C2I (1∶2,000) for 15 min at 4°C to remove non-internalized C2I and C2I fusions, washed again and lysed. Lysates were incubated for 30 min at 37°C with C2I (300 ng) and biotin-labelled NAD+ (10 µM) to ADP-ribosylate actin, which was not ADP-ribosylated by the toxins in the intact cells. Samples were subjected to SDS-PAGE, blotted and biotinylated (i.e. ADP-ribosylated) actin was detected with streptavidin-peroxidase. Comparable amounts of total protein in the lanes were confirmed by Ponceau S staining and Western blot analysis of Hsp90. B. Morphology of the cells described in A after 6 h. C. HeLa cells were incubated with C3bot1E174Q-C2I (4 µg/mL)+C2IIa (8 µg/mL) or with C3bot1E174Q-C2I alone (4 µg/mL). For control cells were left untreated or were incubated with C2I alone (4 µg/mL). After 6 h of incubation at 37°C all cells were washed, incubated with an antibody against C2I for 5 min at 4°C to remove non-internalized C2I and C2I fusions, washed again and pictures were taken. (TIF) [file pone.0054517.s002.tif]

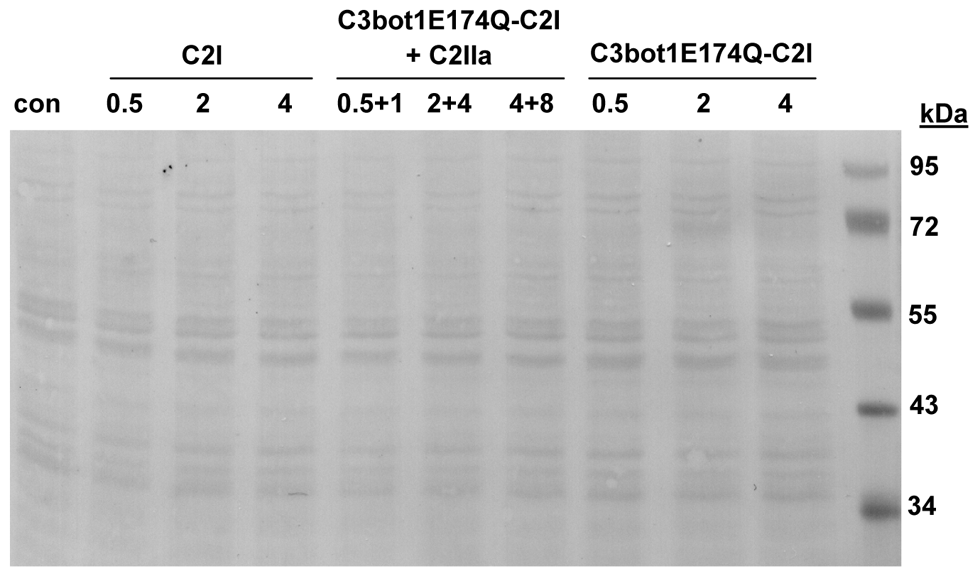

Supplement: Figure S3 — Comparable protein loading in the experiment shown in Fig. 3A was confirmed by Ponceau S staining of the blot membrane. (TIF) [file pone.0054517.s003.tif]
